# Research advances in mesenchymal stem cells and related therapies for rotator cuff tendon-to-bone healing

**DOI:** 10.3389/fbioe.2025.1647909

**Published:** 2025-08-18

**Authors:** Laimeng Song, Na Li, Jiwu Chen

**Affiliations:** ^1^ School of Health Science and Engineering, University of Shanghai for Science and Technology, Shanghai, China; ^2^ Department of Nursing, Huashan Hospital, Fudan University, Shanghai, China; ^3^ Department of Sports Medicine, Shanghai General Hospital, Shanghai, China

**Keywords:** mesenchymal stem cells, rotator cuff tendon-bone healing, conditioned medium, exosomes, tissue engineering, scaffolds, regenerative medicine

## Abstract

Rotator cuff tears (RCTs) are a prevalent cause of shoulder dysfunction, with postoperative retearing remaining a significant challenge due to poor tendon-to-bone healing. Mesenchymal stem cells (MSCs), owing to their multipotency, immunomodulatory properties, and diverse tissue sources, have emerged as a promising therapeutic strategy. Current approaches include direct MSC implantation, MSC-laden scaffolds for structural support, and utilization of MSC-derived conditioned medium (CM) or exosomes to enhance regeneration. Clinical studies demonstrate reduced retear rates with MSC-based therapies, yet animal models show inconsistent outcomes, influenced by cell source, delivery methods, and dosage. MSC modifications (e.g., gene editing) and scaffold-based strategies further improve biomechanical strength and fibrocartilage regeneration. Emerging focus on MSC secretome, particularly exosomes, highlights their potential in modulating inflammation and tissue repair. While preclinical results are encouraging, clinical translation requires standardization of protocols, optimization of delivery systems, and long-term safety evaluations.

## 1 Introduction

Rotator cuff tear (RCT) is a common cause of shoulder pain and dysfunction. It can be caused by acute trauma or chronic overuse, with clinical symptoms mainly including shoulder pain, pain exacerbated by movement, limited range of motion, and muscle atrophy (Teunis et al., 2014; Yamamoto et al., 2010). As the population ages and the number of people participating in sports increases, the incidence of RCT has also risen year by year (Herr et al., 2014). Due to the limited self-healing capacity of rotator cuff tendons, surgery is often required for patients who do not respond to conservative treatments (Dunn et al., 2016; Shin and Lee, 2025; Zingg et al., 2007).

Currently, arthroscopic surgery is the mainstream method for repairing rotator cuff tears. It involves suturing the torn tendon ends back to the bone surface to restore shoulder function (Chen and Chen, 2013; Garcia et al., 2024; Yong, 2018). Despite continuous improvements in surgical techniques and equipment, some patients still experience re-tears after surgery, with the size of the RCT directly affecting the re-tear rate (Bishop et al., 2006; Bjornsson et al., 2011; Sears et al., 2015; Tosyali et al., 2024; Lin et al., 2019). A key factor in this phenomenon is the poor tendon-to-bone healing capacity at the site of the tear (Hernigou et al., 2014; Hernigou et al., 2015; Gupta et al., 2013). Although surgery can reattach the torn rotator cuff to the footprint area, the self-healing ability at the tendon-bone interface is limited. As a result, only loose connective tissue is formed postoperatively, and the sutured rotator cuff tissue cannot regain its original mechanical strength (Weeks et al., 2014).

The native tendon-to-bone interface of the rotator cuff is composed of four distinct layers: bone, mineralized fibrocartilage, unmineralized fibrocartilage, and tendon (Genin and Thomopoulos, 2017; Rossetti et al., 2017). The mineralized and unmineralized fibrocartilage forms a transitional zone that reduces the stiffness gradient between different tissues (bone and tendon), thus buffering mechanical stress and transferring it from the tendon to the bone (Genin and Thomopoulos, 2017; Rossetti et al., 2017). However, this structure does not regenerate after RCT repair, and is instead replaced by fibrovascular scar tissue rich in type III collagen, rather than fibrocartilage, leading to a substantial decrease in biomechanical strength compared to the normal footprint (Hernigou et al., 2014; Hernigou et al., 2015; Gupta et al., 2013). Therefore, promoting the regeneration of the transitional structure at the tendon-to-bone interface and restoring the normal structure of the tendon-to-bone attachment are critical to preventing re-tear after rotator cuff repair.

Mesenchymal stem cells (MSCs) are a class of stem cells with strong proliferative ability and multipotent differentiation potential. They can differentiate into myocytes, osteoblasts, adipocytes, chondrocytes, and other cell types (Polymeri et al., 2016). MSCs are easy to obtain, and can be extracted from bone marrow, tendons, skin, adipose tissue, umbilical cord, blood, and amniotic tissue (Robey, 2017). Their diverse functions include immune modulation, anti-inflammatory effects, anti-apoptosis, and promotion of angiogenesis, making them ideal candidates for tissue engineering research (Cao et al., 2018; Qi et al., 2019; Chen et al., 2023).

In recent years, many researchers have employed various methods to promote tendon-to-bone healing, reduce re-tears, and enhance the biomechanical strength of the new tendon-to-bone attachment, including platelet-rich plasma (Bissell et al., 2015; Spindler et al., 2009; Yang et al., 2017; Gupta et al., 2013), growth factors (Anderson et al., 2001; Huang et al., 2020), gene transfection technologies (Majewski et al., 2008; Zhu et al., 2014), and cell therapy (Huang et al., 2020; Yuan et al., 2025; Sekiya et al., 2015; Valencia et al., 2015; Xiao et al., 2024; Kawai et al., 2015). Among these, MSC-based therapies have shown increasing clinical potential. This article reviews the current research on the application of MSCs in promoting rotator cuff tendon-to-bone healing.

## 2 Application of MSCs and related therapies in rotator cuff tendon-to-bone healing

MSCs can be sourced from several tissues. Bone marrow-derived mesenchymal stem cells (BMSCs) are the most commonly used stem cells and can differentiate into musculoskeletal system cells such as tendon, cartilage, and ligaments under appropriate conditions (Caplan, 1994; Cai et al., 2023). However, bone marrow extraction is painful and may lead to complications (Hjortholm et al., 2013). Another commonly used source is adipose tissue-derived mesenchymal stem cells (ADSCs), which have strong proliferative and differentiation abilities, and their extraction involves less surgical invasiveness compared to BMSCs (Park et al., 2013; Valenzuela et al., 2013). Additionally, synovium-derived mesenchymal stem cells (SDSCs) have recently been discovered (De Bari et al., 2001) and shown to promote cartilage regeneration (Sekiya et al., 2015).

**FIGURE 1:**
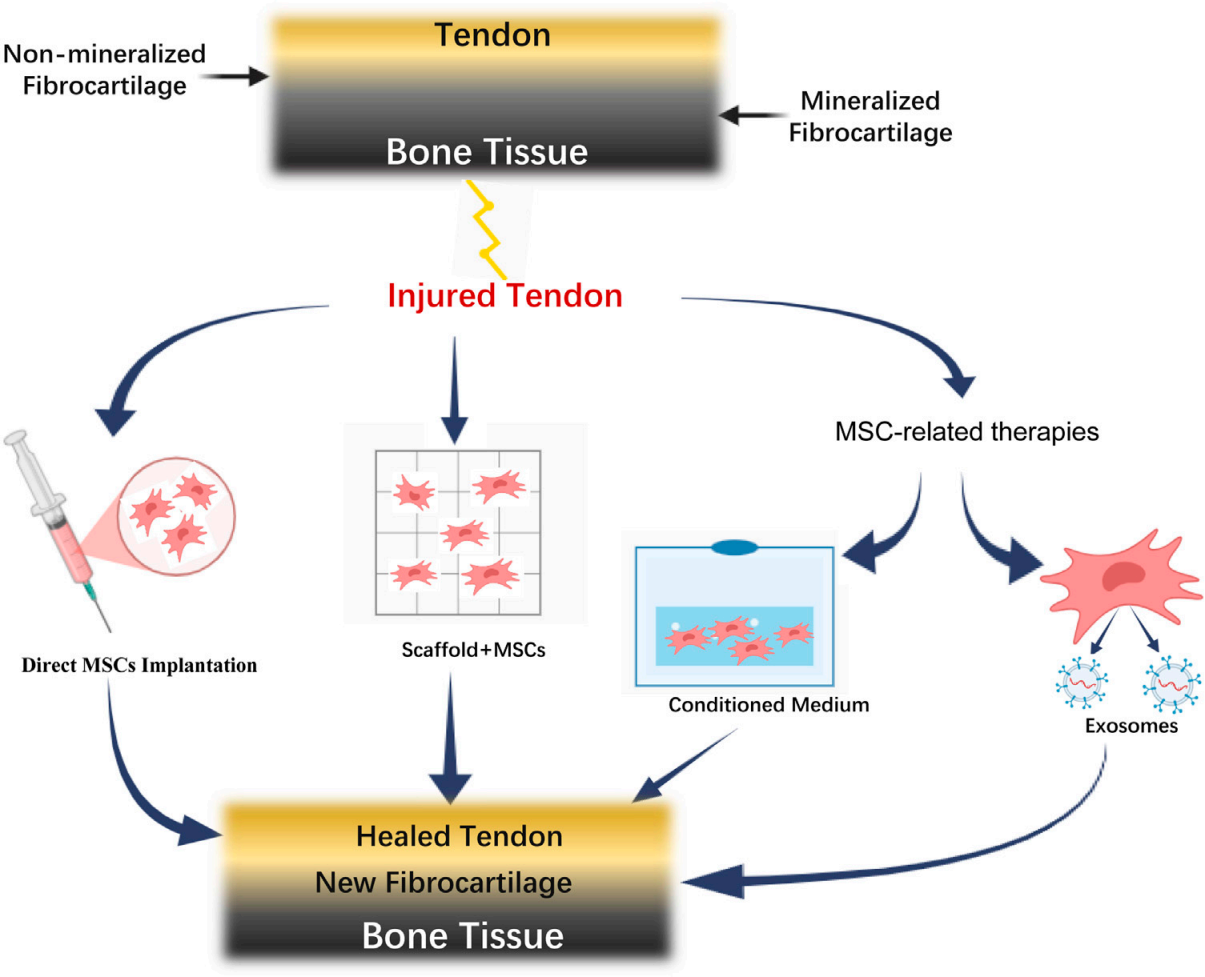
Schematic diagram of MSCs and related therapies for rotator cuff tendon-to-bone healing.

The application of MSCs and related therapies in rotator cuff tendon-to-bone healing involves multiple therapeutic strategies. As illustrated in Figure 1, the normal tendon-bone interface consists of four distinct layers: tendon, non-mineralized fibrocartilage, mineralized fibrocartilage, and bone tissue. Following injury, various MSC-based therapeutic approaches can be employed, including direct MSC implantation, MSC-scaffold combination, and MSC-related therapies such as conditioned medium and exosomes. These strategies ultimately converge to promote tendon-bone healing, resulting in the formation of new fibrocartilage and restoration of the tendon-bone interface structure.

### 2.1 Direct implantation of MSCs

Several clinical studies have reported that MSCs can significantly promote tendon-to-bone healing. Kim et al. explored the effects of ADSCs on recovery in patients after rotator cuff repair. The researchers injected ADSCs, mixed with fibrin glue, into the tendon-to-bone interface and followed up for 28 months. They found that, compared to the control group, although ADSC implantation did not significantly improve shoulder function scores, the re-tear rate in the ADSC group was 14.3%, significantly lower than the control group’s 28.5% (Kim et al., 2017). Hernigou et al. (2014) conducted a 10-year follow-up case-control study and found that in the BMSC treatment group, 39 out of 45 patients (87%) did not experience a re-tear of the rotator cuff, while only 20 out of 45 patients (44%) in the control group-maintained rotator cuff integrity. Furthermore, they divided the patients in the BMSC group into two subgroups based on whether re-tears occurred, and found that the surgical cell implantation dose in the re-tear subgroup was significantly lower than in the non-re-tear subgroup. Thus, they concluded that BMSCs can promote tendon-to-bone healing in the rotator cuff, and this ability is related to the cell implantation dose.

However, the efficacy of MSCs in animal experiments has been inconsistent. Gulotta et al. implanted 10^6 BMSCs into rat rotator cuff repair sites, but found that the implanted MSCs did not improve the histological morphology or biomechanical strength of the tendon-to-bone interface (Gulotta et al., 2009). Degen et al. also found that while ADSC implantation resulted in more organized collagen and better biomechanical strength at the tendon-to-bone interface after 2 weeks, the effects diminished by the fourth week (Degen et al., 2016). The results of various clinical trials and animal experiments mentioned above suggest that although MSCs have the potential to promote tendon-to-bone healing in the rotator cuff, this ability is influenced by several factors, such as the source of the cells, the implantation quantity, and the choice of animal models. Therefore, the treatment protocols still need further exploration.

In addition to directly implanting MSCs into the tendon-bone interface, some researchers have also conducted targeted gene editing of MSCs and injected them into the tendon-bone interface, achieving partial success in animal experiments. Scx is a crucial transcription factor in tendon formation. Gulotta et al. utilized adenovirus-mediated Scx gene delivery into BMSCs and implanted them at the rat rotator cuff tendon-bone interface. Histological and biomechanical analysis revealed that this stem cell approach significantly increased the biomechanical strength of the rotator cuff tendon-bone junction, promoted cartilage formation at the tendon-bone interface, and restored the native fibrocartilage structure (Gulotta et al., 2011).

According to current evidence, transforming growth factor-β (TGF-β) and platelet-derived growth factor-BB (PDGF-BB) are important cytokines that promote tendon-bone healing (Tokunaga et al., 2015; Kovacevic et al., 2015; Yoon et al., 2018; Rieber et al., 2025; Wang et al., 2023). Among these, TGF-β intracellular signaling activity can be inhibited by TGIF1 (Zhang et al., 2013). Therefore, Li et al. used siRNA to knockdown TGIF1 in BMSCs and implanted them into a rat rotator cuff model. The results showed that the biomechanical strength of the newly formed rotator cuff tendon-bone junction in this group was significantly higher than that of the conventional BMSC group or Non-implanted cell group, and the junction morphology was more regular with enhanced cartilage formation (Li et al., 2015). Wang et al. directly upregulated PDGF-BB expression in BMSCs and found that implantation of these modified BMSCs resulted in a significant increase in the maximum tensile strength of the newly formed rat rotator cuff tissue compared to the simple BMSC group or the non-cell-implanted group (Wang LL. et al., 2018). Other studies have also enhanced osteogenic potential and proliferative activity of BMSCs by knocking out the TOB1 gene, further promoting tendon-bone healing in rat rotator cuff models.

### 2.2 MSCs combined with tissue engineering approaches

In the aforementioned studies, MSCs are typically dissolved in a gel matrix and injected into the local area of the rotator cuff. Although this method has shown some efficacy, it cannot guarantee the retention of cells at the local site after injection, as the cells are prone to diffuse into the tissue gaps, which not only affects the therapeutic effect but may also cause side effects (Hernigou et al., 2014; Chen et al., 2022; Hutmacher, 2000). Given that the tendon-bone interface of the rotator cuff is not a closed environment, the cell injection technique alone cannot fully meet the requirements for rotator cuff tendon-bone insertion reconstruction.

On the other hand, for more complex RCTs, Neviaser et al. proposed the use of grafts as scaffolds to fill the defects (Neviaser et al., 1978). Since then, various types of grafts (such as autografts, allografts, synthetic grafts, and xenografts) have been gradually applied to treat large, irreparable RCTs, achieving some success (Wang LL. et al., 2018; Han et al., 2019; Gupta et al., 2013). On the other hand, artificial synthetic materials may cause significant immune reactions post-surgery, whereas biological materials, despite having a smaller risk of rejection and being degradable, may not fully meet the mechanical properties required for rotator cuff function (Shang et al., 2014; Ye and Bao, 2015). Lin et al. found through a systematic review that the re-tear rate after repair with graft patches for massive rotator cuff tears can still reach 4.5%–55% (Lin et al., 2019).

Given that grafts can provide an adhesive environment for local cells and stem cells have strong regenerative potential, an increasing number of researchers are using scaffolds loaded with MSCs to treat tendon-bone healing after rotator cuff tears. Scaffolds ensure the uniform delivery of cells to the target area, enhancing the retention and survival rates of stem cells while providing three-dimensional support for tissue regeneration (Hutmacher, 2000).

Kim et al. (2013) applied Polylactic Acid Scaffold loaded with BMSCs in a rabbit model acute rotator cuff repair. Over the following 6 weeks, a large number of BMSCs were observed to survive, and the collagen I-positive areas in the BMSC-loaded scaffolds were significantly higher than in the plain scaffold. Yokoya et al. used polycaprolactone (PCL) scaffolds loaded with BMSCs to treat large rotator cuff tears in rabbits acute. They found that, compared to scaffolds without MSCs, the tendon-bone insertion site in the BMSC-loaded group showed newly formed fibrocartilage at 8 weeks, significantly improving the biomechanical strength of the regenerated tissue (Yokoya et al., 2012). Thangarajah et al. created a decalcified cortical bone scaffold, which, when combined with BMSCs, successfully promoted tendon-bone healing in a rat rotator cuff model (Thangarajah et al., 2018). Furthermore, some researchers used transgenic BMSCs combined with 3D-printed poly-lactic-co-glycolic acid (PLGA) scaffolds to promote tendon-bone healing in a rabbit rotator cuff model. They found that this approach improved collagen alignment in the Freshman tissue and increased the amount of fibrocartilage formation (Chen P. et al., 2019). In terms of longer-term outcomes, Dai et al. developed dual cross-linked COL1/HAp bionic gradient scaffolds loaded with human amniotic mesenchymal stem cells (hAMSCs) and evaluated their effects in a rat rotator cuff model. Their results at 12 weeks post-operation demonstrated that the hAMSC-loaded scaffolds significantly enhanced tendon-bone interface healing with excellent collagen fiber continuity and orientation, increased fibrocartilage and bone formation, and markedly improved biomechanical properties compared to the control group, providing valuable insights into the long-term efficacy of MSC-scaffold combinations for rotator cuff repair (Dai et al., 2024).

It can be said that current research on stem cells combined with scaffolds to promote tendon-bone healing of the rotator cuff has yielded promising results in animal models. In the future, it is necessary to investigate whether this strategy has the same efficacy in humans and to identify suitable scaffolds and corresponding loading strategies to enhance the effectiveness of stem cell-based repair for rotator cuff tears.

### 2.3 MSCs-related therapies

In recent years, studies have found that although bone marrow mesenchymal stem cells (BMSCs) may not differentiate into the corresponding cells of target organs *in vivo*, they can still exert therapeutic functions. Further research has shown that these effects are mediated by their secretome (Xiaoli et al., 2018; Liu et al., 2020). The secretome contains various nutritional factors secreted by mesenchymal stem cells (such as chemokines, cytokines, growth factors, hormones, and lipid mediators) as well as vesicular substances, and these components can affect neighboring cells (Xiao et al., 2024; Kawai et al., 2015; Wang et al., 2024; El Moshy et al., 2020). Based on this, the application of the secretome in sports medicine has gradually attracted attention, and some studies have applied it to promote tendon-bone healing. However, the clinical application of mesenchymal stem cells (MSCs) is somewhat limited due to their potential tumorigenicity and ethical concerns. The primary issue is tumorigenicity, because mesenchymal stem cells have self-renewal capacity and may undergo malignant transformation under certain conditions (Motaln et al., 2010). Although MSCs themselves are generally considered non-tumorigenic, their long-term fate after implantation and potential genetic instability remain areas of active investigation by researchers. Studies have shown that MSCs can promote tumor growth through paracrine effects, angiogenesis stimulation, and immune modulation, especially in the presence of pre-existing malignancies. Ethical issues surrounding the sources of stem cells also require careful consideration, including issues related to tissue commercialization, informed consent, and donor site morbidity.

### 2.4 MSCs conditioned medium

Conditioned medium (CM) refers to the culture medium that contains various substances released by the cell population in the culture dish after a period of *in vitro* cultivation (Bogatcheva and Coleman, 2019; Pawitan, 2014). It is easy to collect, convenient for storage and transportation, has no immunogenicity, and can be frozen and dried. These advantages provide a foundation for its clinical application (Bogatcheva and Coleman, 2019).

MSCs-derived CM has various promoting effects. It has been found to promote stem cell proliferation and enhance their osteogenic capacity (Xiao et al., 2024; Kawai et al., 2015; Wang et al., 2024; An et al., 2013), induce pluripotent stem cells to differentiate toward chondrogenesis (Lee et al., 2014), and work synergistically with TGF-β to improve the collagen secretion ability of fibroblasts (Lee et al., 2014). Based on this, researchers have applied MSCs-derived CM to promote tendon-bone healing and have made some progress.

Sun et al. collected BMSCs-derived CM and injected it into the joint cavity of a rat model after anterior cruciate ligament reconstruction. They found that, compared to rats injected with DMEM culture medium or those that received no injection, the CM group showed less fibrous scar tissue between the graft and bone tunnel at 4 and 8 weeks. Additionally, more Sharpey’s fibers were generated, and the mechanical strength of the graft in the joint cavity segment was also enhanced, with a more organized collagen arrangement (Sun et al., 2019). Chen et al. created an arthritis model in rats by inducing cruciate ligament rupture and subsequently found that intra-articular injection of CM could protect articular cartilage and delay the progression of arthritis (Chen W. et al., 2019). Sevivas et al. discovered that BMSCs-derived CM could enhance tendon cell proliferation, and when the stimulated cells were implanted into a rat rotator cuff repair model, they significantly increased the biomechanical strength of the newly formed tendon-bone junction, indirectly confirming the function of CM (Sevivas et al., 2018). Regarding long-term follow-up, Dai et al. also confirmed that dual cross-linked gradient COL1/HAp scaffolds loaded with human amniotic mesenchymal stem cells facilitated rotator cuff healing in rats model at 12 weeks post-operatively, demonstrating excellent continuity and orientation of collagen fibers, increased fibrocartilage formation, and significantly improved biomechanical properties at the tendon-bone interface (Dai et al., 2024).

In summary, CM derived from MSCs indeed holds the potential to promote rotator cuff tendon-bone healing. Future research needs to clarify whether the functions of CM derived from different MSCs sources vary, how to optimize the composition of CM to enhance its ability to promote tendon-bone healing, and to identify suitable carriers for CM, while also evaluating the safety of this therapy.

### 2.5 MSCs exosomes

Exosomes are small secretory vesicles with a diameter of 30–150 nm and serve as one of the mediators of intercellular communication. They can transfer bioactive lipids, nucleic acids, and proteins between cells, thereby mediating various biological functions of recipient cells (Bruno et al., 2017). Exosomes derived from MSCs have the ability to promote tissue regeneration, regulate the local immune environment, and have been shown to exert therapeutic effects in animal models of myocardial infarction, stroke, limb ischemia, perinatal hypoxic-ischemic brain injury, kidney injury, and osteochondral injury (Pawitan, 2014; Bruno et al., 2017; Liu, 2019; Miao et al., 2019; Zhu et al., 2018).

Currently, there are no reports on the application of exosomes in tendon-bone healing, but there is considerable evidence indicating that MSCs-derived exosomes can be used in the treatment of musculoskeletal diseases.

For example, MSCs-derived exosomes can significantly enhance bone mineral density in osteoporotic rats (Qi et al., 2016; Zhang et al., 2020). When MSCs are induced to undergo osteogenic differentiation, the exosomes they produce also exhibit osteogenic effects (Wang X. et al., 2018). Furthermore, MSCs-derived exosomes have been shown to promote cartilage regeneration. Cosenza et al. reported that MSC-derived exosomes, while inhibiting catabolic and inflammatory markers, reinduce the expression of cartilage matrix, protecting articular cartilage (Cosenza et al., 2017). Moreover, exosomes play a beneficial role in tendon injury and repair. Shen et al. found that MSCs-derived exosomes can modulate macrophage polarization, thereby altering the local inflammatory environment and promoting tendon regeneration (Shen et al., 2020). Yu et al. also discovered that MSCs-derived exosomes can promote the proliferation and migration of tendon stem cells and mediate their differentiation into tendon cells (Yu et al., 2020).

Due to the carrier properties of exosomes, current research also explores the use of different interventions to MSCs to obtain exosomes with distinct contents, thereby exerting various biological functions. For example, overexpression of miR-140-5p inside MSCs can result in exosomes enriched with miR-140-5p, and these exosomes enhance the proliferative capacity of chondrocytes, thereby protecting cartilage (Tao et al., 2017). Mao et al. used the same method to obtain MSC-derived exosomes enriched with miR-92a-3p, finding that these exosomes have chondrogenic effects (Mao et al., 2018). Li Chaofu et al. applied hypoxic stimulation to MSCs to obtain exosomes with high expression of miR-214, and found that these exosomes exert cardioprotective effects (Chaofu et al., 2019).

Based on the above studies, it is evident that MSC-derived exosomes possess the ability to promote osteogenesis, chondrogenesis, and tendonogenesis, indicating their potential to facilitate tendon-bone healing. Future research can focus on areas such as the effective concentration of exosomes, the key components of their contents, and how to regulate the exosomal contents to enhance their regenerative functions.

## 3 Conclusion and future directions

While the therapeutic potential of MSCs in rotator cuff tendon-to-bone healing is promising, it is crucial to address the safety concerns associated with their clinical application. The issues of tumorigenicity, immunogenicity, and ethical considerations must be carefully evaluated and managed through strict quality control measures, appropriate cell source selection, and adherence to established regulatory guidelines. Future research should focus on developing safer delivery methods, optimizing cell dosages, and establishing long-term safety monitoring protocols to ensure the successful clinical translation of MSC-based therapies.

Reducing the occurrence of re-tear after rotator cuff repair has been a research focus in both the field of sports medicine and regenerative medicine. The implantation of MSCs and related therapeutic strategies (such as using scaffolds, or collecting their CM or exosomes) have provided various approaches for rotator cuff tendon-bone healing. In the future, MSC-related treatment plans can be optimized, or untested methods can be validated.
